# Need for Scientific Rigor in the Evaluation of Minimally Invasive Alternative Procedures

**DOI:** 10.1155/2015/876496

**Published:** 2015-04-28

**Authors:** Johnny Padulo, Luca Paolo Ardigò

**Affiliations:** ^1^eCampus University, Via Isimbardi, No. 10, 22060 Novedrate, Italy; ^2^Tunisian Research Laboratory “Sports Performance Optimization”, National Center of Medicine and Science in Sport, 263 Tunis, Tunisia; ^3^School of Exercise and Sport Science, Department of Neurological and Movement Sciences, University of Verona, Via Felice Casorati, No. 43, 37131 Verona, Italy

This is a comment on “One-Year Follow-Up of a Series of 100 Patients Treated for Lumbar Spinal Canal Stenosis by Means of HeliFix Interspinous Process Decompression Device” [1]. We read a recent article [1] on a conservative treatment in 100 patients as a formidably minimally invasive method to improve quality of life. We think that this article presents (supported by a tremendous number of both patients and references) an innovative approach worthy of scientific research [2]. Nevertheless, several points listed in this letter point out what is yet necessary to verify [3] for the sake of treatment effectiveness and patients' safety. Particularly methodological approach shows some flaws [4–6], which lead to unclear results interpretation.

Therefore, this letter aims to help the reader to better understand the treated matter [3].

In Section 2, the authors state the following:

- In Section 2.1, Table 1 should provide information about the amount of variation of age (e.g., as standard deviation) [7]. Such information is not disclosed neither in text nor in Table 1. Such a relevant sample size would deserve that kind of information.
- In Section 2.3, paragraph “…walking condition and distance…”, this information is rather relevant, as well. Yet gait and covered distance assessments (e.g., which walking and/or walking fitness test has been used?) have not been described [8]. And, most of all, precise figures are missing: “…patients with IPDs implanted were judged by their physician to walk ‘fluently.'” represents surely an informed opinion but cannot satisfy curiosity of researcher outside the neurosurgeons circle. Table 2 cannot do that either, at least about walking assessment: “Patients were visited monthly in the first three months, and again at 6 and at 12….” Five assessments require a specific statistical analysis, that is, ANOVA for repeated measures [9].

Surely, we agree that any kind of minimally invasive alternative procedure should be operated to preserve spinal stenosis patients' quality of life. And we acknowledge that interspinous devices can be applied safely and effectively in selected patients, as well. Yet this study lacks both an adequately long-term analysis and a proper statistical analysis. Sound research on the specific matter needs to be performed in advance before applying any kind of treatment most of all for effective patients' long-term satisfaction [10].

## References

[1] Alexandre A., Alexandre A. M., de Pretto M., Corò L., Saggini R. (2014). One-year follow-up of a series of 100 patients treated for lumbar spinal canal stenosis by means of HeliFix interspinous process decompression device. *BioMed Research International*.

[2] Padulo J., Ardigò L. P. (2014). Evaluating BCI devices: a statistical perspective. *Ergonomics*.

[3] Padulo J., Maffulli N., Ardigò L. P. (2014). Signal or noise, a statistical perspective. *Proceedings of the National Academy of Sciences of the United States of America*.

[4] Huang D.-G., He S.-M., Pan J.-W. (2014). Is the 4 mm height of the vertebral artery groove really a limitation of C1 pedicle screw insertion?. *European Spine Journal*.

[5] Padulo J., Ardigò L. P. (2014). Formetric rasterstereography: a new perspective. *Osteoporosis International*.

[6] Padulo J., Oliva F., Ardigò L. P. (2014). Letter to the Editor concerning ‘Calculation of corrected body height in idiopathic scoliosis: comparison of four methods’ by M. Tyrakowski et al. (Eur Spine J, doi:10.1007/s00586-014-3275-1). *European Spine Journal*.

[7] Atkinson G., Nevill A. M. (1998). Statistical methods for assessing measurement error (reliability) in variables relevant to sports medicine. *Sports Medicine*.

[8] Winter E. M. (2012). Calibration and verification of instruments. *Journal of Sports Sciences*.

[9] Hopkins W. G., Marshall S. W., Batterham A. M., Hanin J. (2009). Progressive statistics for studies in sports medicine and exercise science. *Medicine and Science in Sports and Exercise*.

[10] Lee K. P., Boyd E. A., Holroyd-Leduc J. M., Bacchetti P., Bero L. A. (2006). Predictors of publication: characteristics of submitted manuscripts associated with acceptance at major biomedical journals. *Medical Journal of Australia*.

